# Lung and eye involvement in X-linked hypohidrotic ectodermal dysplasia

**DOI:** 10.1186/1746-160X-8-S1-P3

**Published:** 2012-05-25

**Authors:** J Dietz, T Kaercher, AT Schneider, T Zimmermann, K Huttner, R Johnson, H Schneider

**Affiliations:** 1 Department of Pediatrics, University Hospital Erlangen, Germany; 2 Edimer Pharmaceuticals Inc, Cambridge, USA

## Objective

X-linked hypohidrotic ectodermal dysplasia (XLHED; ectodysplasin deficiency) has been classically described as affecting hair, sweat glands and dentition. What may be underappreciated is the effect ectodysplasin deficiency has on glands surrounding the airways and eyes and the resulting chronic health issues. In this study, we evaluated respiratory and ocular symptoms in XLHED patients.

## Study design

12 male children and 14 male adults with XLHED, age range 6 to 58 years, and 12 healthy controls were assessed for signs of asthma by pulmonary function tests and measurement of exhaled nitric oxide (FeNO), and for dry eye disease by investigating ocular surface lubrication. Descriptive statistics were calculated. Standardized sweat duct counts and *EDA* genotype were included in correlation analyses.

## Results

Respiratory symptoms and elevated FeNO as a sign of pulmonary inflammation were detected in the majority of XLHED subjects, in similar numbers of children and adults. Increased tear osmolarity, reduced tear film break-up time and other ocular abnormalities were also present at an early age. Approximately half of the patients not reporting a history of asthma or dry eye showed at least two abnormal test results in the respective organ system. The presence of residual sweat ducts, suggestive of partial *EDA* gene expression, correlated with milder disease in two XLHED subjects with mutations affecting the collagen-like domain of ectodysplasin.

## Conclusions

The high prevalence of asthma-like symptoms and dry eye syndrome in XLHED patients as young as 6 years indicates that screening evaluation, regular monitoring and consideration of therapeutic intervention should begin in early childhood.

